# Clinicians’ Perspective on Implementing Virtual Hospital Care for Low Back Pain: Qualitative Study

**DOI:** 10.2196/47227

**Published:** 2023-11-21

**Authors:** Alla Melman, Simon P Vella, Rachael H Dodd, Danielle M Coombs, Bethan Richards, Eileen Rogan, Min Jiat Teng, Chris G Maher, Narcyz Ghinea, Gustavo C Machado

**Affiliations:** 1 Sydney Musculoskeletal Health The University of Sydney and Sydney Local Health District Camperdown Australia; 2 The Daffodil Centre Faculty of Medicine and Health a joint venture between The University of Sydney and Cancer Council New South Wales Sydney Australia; 3 Physiotherapy Department Royal Prince Alfred Hospital Sydney Australia; 4 Rheumatology Department Royal Prince Alfred Hospital Sydney Australia; 5 Canterbury Hospital Sydney Australia; 6 RPA Virtual Hospital Sydney Australia; 7 Department of Philosophy Macquarie University Sydney Australia

**Keywords:** back, barrier, barriers, care model, eHealth, e-Health, enabler, enablers, facilitator, facilitators, health services research, healthcare delivery, implementation, interview, interviews, low back pain, model of care, pain, qualitative, remote care, service delivery, service, services, telehealth, telemedicine, virtual care, virtual hospital

## Abstract

**Background:**

Alternate “hospital avoidance” models of care are required to manage the increasing demand for acute inpatient beds. There is currently a knowledge gap regarding the perspectives of hospital clinicians on barriers and facilitators to a transition to virtual care for low back pain. We plan to implement a virtual hospital model of care called “Back@Home” and use qualitative interviews with stakeholders to develop and refine the model.

**Objective:**

We aim to explore clinicians’ perspectives on a virtual hospital model of care for back pain (Back@Home) and identify barriers to and enablers of successful implementation of this model of care.

**Methods:**

We conducted semistructured interviews with 19 purposively sampled clinicians involved in the delivery of acute back pain care at 3 metropolitan hospitals. Interview data were analyzed using the Theoretical Domains Framework.

**Results:**

A total of 10 Theoretical Domains Framework domains were identified as important in understanding barriers and enablers to implementing virtual hospital care for musculoskeletal back pain. Key barriers to virtual hospital care included patient access to videoconferencing and reliable internet, language barriers, and difficulty building rapport. Barriers to avoiding admission included patient expectations, social isolation, comorbidities, and medicolegal concerns. Conversely, enablers of implementing a virtual hospital model of care included increased health care resource efficiency, clinician familiarity with telehealth, as well as a perceived reduction in overmedicalization and infection risk.

**Conclusions:**

The successful implementation of Back@Home relies on key stakeholder buy-in. Addressing barriers to implementation and building on enablers is crucial to clinicians’ adoption of this model of care. Based on clinicians’ input, the Back@Home model of care will incorporate the loan of internet-enabled devices, health care interpreters, and written resources translated into community languages to facilitate more equitable access to care for marginalized groups.

## Introduction

### Overview

In Australia, back pain is the fifth most common reason for emergency department (ED) visits and ranks third for those aged between 40 and 69 years [1]. About one-third of patients presenting to the ED with back pain are subsequently admitted to the hospital, staying an average of 9 days [2]. Not only is hospital admission for back pain costly (US $10,000 per admission), but it also contributes to increased patient morbidity and a delay in recovery time [3]. With an increasingly aging population [4], hospital inpatient admissions have continued to steadily rise, while the number of available beds has consistently reduced [5].

Alternate “hospital avoidance” models of care are required to manage the increasing demand on acute inpatient beds by facilitating hospital-level health care delivery for patients in their own place of residence. “Hospital in the home” models of care are now well established worldwide and are associated with decreased length of stay and increased patient and caregiver satisfaction [6,7]. A recent evolution of these models is the “virtual hospital,” such as the one recently implemented by the Sydney Local Health District (rpavirtual) [8].

We plan to implement a virtual hospital model of care for back pain called “Back@Home” and to use qualitative interviews with stakeholders to develop and refine the model. The “Knowledge to Action Framework” [9] encourages clinicians’ involvement in developing evidence-based solutions for health care delivery while adapting these to the local context. This approach to the research cycle helps to address barriers to implementation, optimizing successful clinician engagement and care delivery [10].

A cohort study comparing more than 15,000 in-person consults to over 5000 telehealth consults for acute low back pain in primary care found that telehealth was associated with a lower rate of referral for lumbar imaging [11] while yielding comparable quality performance measures. This finding suggests that the provision of high-value, low back pain care through telehealth is possible.

There is currently a knowledge gap regarding the perspectives of hospital clinicians on reasons for hospital admission for acute back pain, as well as their perspectives on barriers and facilitators of a transition to virtual care for this condition. This study will have a significant impact on the successful implementation of a novel virtual hospital model of care (Back@Home). By influencing hospital policy and procedures, it will also potentially positively affect clinical outcomes for hospital back pain presentations.

### Objective

The objective of this study was to explore clinicians’ perspectives on a virtual model of care for back pain and identify barriers to and enablers of successful implementation of this new model of care.

## Methods

### Ethical Considerations

This study was approved by the Sydney Local Health District (LHD) Human Research Ethics Committee (X21-0094). Participants provided informed consent after reading the participant information sheet and being given an opportunity to ask questions. All study data was stored on a password-secured server. No compensation was provided for participants.

### Study Design, Setting, and Recruitment

This study was a qualitative study using semistructured interviews conducted from June to December 2021.

Participants were recruited from 3 metropolitan hospitals in Sydney LHD: Royal Prince Alfred Hospital, Concord Repatriation General Hospital, and Canterbury Hospital, through purposive sampling. Sydney LHD has approximately 400 admissions per year, with a primary admission reason of musculoskeletal low back pain [12,13] and an ED admission rate of nearly 17% [14].

The inclusion criteria were as follows: clinicians (physicians, physiotherapists, occupational therapists, social workers, and nurses) employed in Sydney LHD who had experience managing people presenting with low back pain. All clinicians involved in the management of low back pain in Sydney LHD were invited to participate in initial interviews by email and at staff meetings. All participants were provided with a participant information statement and provided written consent to participate. Participants were able to withdraw their consent at any stage, up until the data had been analyzed.

Participants were not familiar with the interviewer before the commencement of the study. An interview guide (Multimedia Appendix 1) provided structure but allowed scope for additional questions as the interview progressed. The interview guide was developed to explore the key barriers and enablers to virtual hospital care and was piloted with 3 participants. An experienced qualitative researcher (RD) then reviewed the recordings and provided feedback on improvements to the structure and delivery of interview questions. Pilot data were included in the final analysis.

Semistructured interviews were conducted through videoconferencing (Zoom; Zoom Video Communications) or telephone, lasting up to 30 minutes. Interviews were digitally audio recorded, transcribed verbatim, and then proofread. Recruitment for participants was discontinued when the research team concluded that no new emerging themes had developed from consecutive interviews [15].

### Analysis

Samples of transcript data (5 interviews) were independently annotated by 4 authors (AM, SV, GCM, and RD), creating a bank of initial ideas and phrases. Emerging concepts were then compared between authors and merged to develop a coding framework of key themes [16]. Themes were identified as significant based on frequency and relevance to the implementation of the model of care. All interviews were then coded by 1 author (AM), using the coding framework that was agreed upon.

Key themes and participant quotes were mapped to subcategories according to the Theoretical Domains Framework (TDF) [17,18]. The TDF [19] provides 14 domains with which to explore the determinants of health care professionals’ behavior change, thus informing implementation strategies. A summary of barriers to and enablers of virtual hospital care from the point of view of clinicians was developed. Demonstrative quotes supporting key themes were identified and used to support the findings.

**Figure 1:**
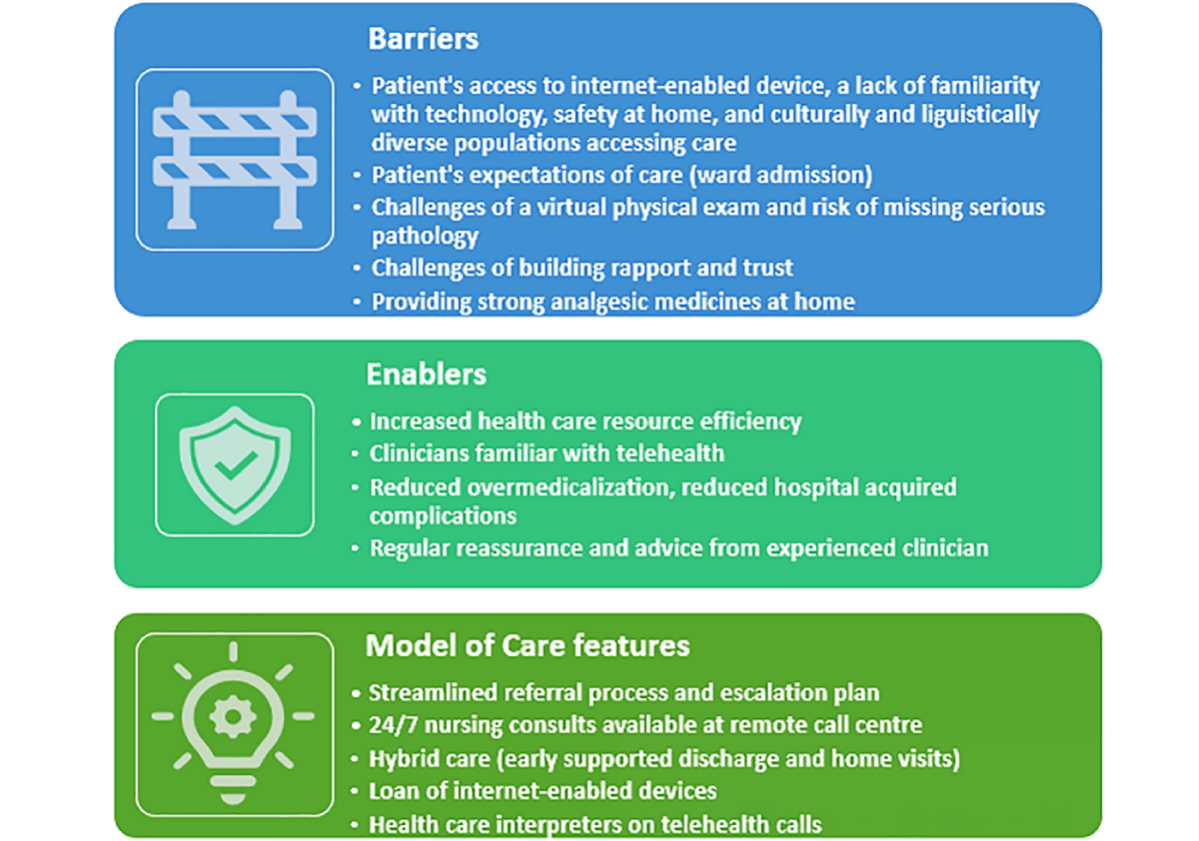
Barriers, enablers, and model of care features suggested by participants.

Data were analyzed using NVivo (QSR International) software and reported according to the COREQ (Consolidated Criteria for Reporting Qualitative Research) checklist [20]. A summary of the findings is provided in Figure 1.

### Reflexivity

Disclosure of researchers’ backgrounds allows the reader to understand how the authors’ viewpoints may have influenced data interpretation. Several researchers have clinical experience working with people experiencing back pain (AM, SV, GCM, CGM, DMC, BR, ER, and MJT). The remaining authors (RD and NG) were involved for research methodology expertise. All interviews were conducted by a single researcher (AM), an experienced musculoskeletal physiotherapist (of more than 18 years) and a PhD student.

## Results

### Participant Characteristics

A total of 19 clinicians participated in this study, of which 9 participants were rheumatology consultants (RC 1-9), with admitting rights to the hospital for patients with low back pain. Another 3 participants were rheumatologists-in-training (trainees; RT 1-3), and 1 was an emergency physician trainee (ET 1). A total of 4 participants were senior physiotherapists (PH 1-3), and 2 were senior emergency nurses (EN 1 and2).

All participants had direct clinical experience managing patients with back pain, either in the ED or as inpatients. These clinicians would likely be involved in referring patients to Back@Home but not delivering virtual hospital care. However, many clinicians at Sydney LHD have been involved in remote care services such as hospital-in-the-home at the rpavirtual hospital since the beginning of the COVID-19 pandemic.

### Thematic Analysis of Clinician Interviews

Insight into barriers and enablers is essential to providing an understanding of the complexities of implementing a novel model of care where there is potential for clinical benefit and a need for due consideration of patient safety. A total of 10 of the 14 key TDFs [17] were identified as being most relevant and important: “Environmental Context and Resources”; “Knowledge”; “Skills”; “Emotion”; “Beliefs about Consequences”; “Reinforcement”; “Social Influences”; “Professional Role and Identity”; “Beliefs about Capabilities”; and “Memory, Attention, and Decision Processes.” TDF dimensions that were not seen in the data or were well covered by a similar theme were not presented (“Goals,” “Optimism,” and “Intentions and Behavioral Regulation”).

### Barriers to a Virtual Model of Care: Resources and Expectations

#### Environmental Context and Resources, Knowledge, and Skills

Perceived barriers to virtual care included patients’ lack of access to internet-enabled devices and a lack of competence in the use of web-based exercise applications and videoconferencing platforms. Clinicians were especially concerned about how telemedicine could change the dynamics of access, potentially amplifying inequities. This was especially true for patients from culturally and linguistically diverse (CALD) communities and older patient cohorts unfamiliar with technology.

I think the barriers for our patients are around social deprivation….people who don’t speak English, people who are uncomfortable with technology, and that will be our older patients who are often the people who will present to hospital.

Consultant rheumatologist, RC5

#### Emotion (Fear, Anxiety, and Stress)

Participants were worried that telehealth treatment would adversely impact the patient-clinician relationship as it is more difficult to establish rapport and trust. This could have consequences for patient outcomes. For instance, physiotherapists and rheumatologists thought that patients would mobilize less without the motivation of face-to-face physiotherapy.

Having that face-to-face interaction establishing rapport…sharing examples of previous patients that have been through similar things and providing that reassurance face to face, comparatively, can be…a little bit more reliable.

Physiotherapist, PH4

#### Knowledge (of Condition), Beliefs About Consequences (Outcome Expectancies), and Reinforcement (of Patient Behavior)

Patient care expectations were noted as a major barrier to virtual care. While clinicians agreed with guideline-based advice [21] that back pain is generally self-limiting, with a good prognosis, and therefore not requiring hospitalization, they noted that many patients expected to receive spinal imaging, pharmacological pain relief, or to be admitted to the hospital.

Managing patients’ care expectations and encouraging proactive self-management were therefore recognized as important roles for frontline clinicians. On this point, a consultant rheumatologist commented that:

[The] expectation is that if they go to the hospital that the hospital will be able to resolve that problem for them, so there’s often quite a complex education piece…to help the patient to understand that with non-serious back pain…the person who’s going to make that better is primarily them. There’s nothing we can do to them that’s going to make them…instantly better.

Consultant rheumatologist, RC5

Hospital staff may therefore find themselves forced to advocate for options the patient finds difficult to accept.

When people are in a significant amount of pain and you’re telling them that physiotherapy and time and analgesia are the answer rather than procedures necessarily. Some people find that difficult to reconcile.

Rheumatologist in-training, RT3

Clinicians explained that if a patient is admitted to the hospital for back pain, it sets a precedent that reinforces the patient’s expectation of being admitted again in the future. To avoid this reinforcement pattern, participants agreed that admissions are best avoided when possible, and care in the community or through hospital outpatient care was preferred.

[It is] counterproductive for people to be admitted to hospital…they’re likely to fall into that same pattern again.

Consultant rheumatologist, RC6

#### Social Influences (Social Support and Alienation) and Emotion (Depression)

Participants described that it can be difficult to discharge patients home from the ED due to concern for their safety and well-being. This was especially the case for patients who had no social support and were not coping at home. In older patients, back pain was recognized as being one aspect of a complex presentation, and the decision to admit them to the hospital would include an assessment of their ability to manage the basic activities of daily living.

Concerns were also raised about sending home patients with mental health comorbidities, such as depression or suicidal ideation related to their pain. An ED nurse described the interplay between severe chronic pain and mental health:

I’ve seen…chronic pain manifest as an acute mental health issue. That represents some risk to the patient….I’ve certainly seen people express suicidal ideations because they’ve been in a state of chronic pain.

Emergency nurse, EN1

### Barriers to a Virtual Model of Care: Medicolegal Concerns

#### Skills, Professional Role and Identity, and Beliefs About Capabilities

Participants had reservations regarding performing a thorough neurological examination virtually, with concerns about missing serious pathology without a face-to-face consult. A physiotherapist shared their concerns regarding identifying serious pathologies such as myelopathy or cauda equina using telehealth.

My concern would be identifying the deteriorating patient. So if they had deteriorating neurological signs…how can we do that, virtually…can we assess their gait? How can we assess that they have now developed a foot drop? Can we assess that their reflexes are changed? What if they’ve got urinary retention? We can’t assess that remotely.

Physiotherapist, PH1

Employing new graduate inexperienced Physios could potentially leave...the model of care open to harm because the inexperienced Physio may miss things that are being told to them”

Physiotherapist, PH2

Other concerns raised included being unable to discharge patients home if they were not mobilizing safely and potential liability if they were to fall at home. A consultant rheumatologist speculated as to the mechanisms in place to protect clinicians should a patient be transferred to virtual care and have a fall at home, specifically:

Whether there’s a legal framework to protect a clinician against...indemnity claims in that circumstance.

Consultant rheumatologist, RC1

A practical concern was how to safely administer analgesia, including opioids, in a virtual care model. Considerations included what types of analgesia to prescribe in virtual care and which patients would be considered fit to manage administration of sedating medication independently. For example:

The patient would have to be savvy enough with their medications to understand…what to do when, not just pop pills regardless. Sometimes we are dishing out quite heavy-duty pain relief to these people to try and get them through.

Consultant rheumatologist, RC3

### Enablers of a Virtual Model of Care: Organizational and Professional

#### Environmental Context and Resources (Organizational Culture)

For hospitals, the prospect of more efficient use of limited health care resources was seen as an important enabler of virtual models of care such as Back@Home. Participants believed these models had the potential to create cost savings by shortening the length of stay, increasing bed availability, and improving patient flow in the ED setting.

From a hospital perspective, especially given the current climate, it certainly frees up bed space and…allows us to ensure good patient flow through so we can…dedicate our workforce to people who...need that care.

Physiotherapist, PH4

There’s really no reason why people can’t be managed in an environment outside hospital.

Consultant rheumatologist, RC8

#### Social and Professional Role and Identity (Professional Identity, Professional Role, and Patients’ Role)

The familiarity that clinicians had developed with telehealth during the pandemic was seen as a key enabler for the ongoing implementation of virtual care models. In addition, clinicians were aware of the advantages of these models, which could help overcome resistance to their implementation. For instance, it was noted that virtual models of care reduced unnecessary hospital admissions and permitted more frequent contact with patients.

I feel like a lot of our care is now becoming virtual…I think that would be great if it would keep them out of hospital…It’ll take a lot of pressure off us to be honest.

Emergency nurse, EN2

The secret is the fact that you’ve got the regular contact. I mean, that’s really what you’re buying in hospital…regular contact with clinicians.

Consultant rheumatologist, RC8

Reduction in unnecessary admissions was seen as particularly advantageous as it could reduce medicalization of low back pain and avoid reinforcing illness behaviors and institutionalization that can contribute to functional decline.

[Admission] might present as a message to the patient that they’re sick...they’re lying in bed, and it’s okay to be in bed, which is not the message we want to give to the patient. It may also generate extra incidental investigations which may not be required otherwise if it was managed in the community.

Consultant rheumatologist, RC9

Patients do get a bit institutionalised…if they’re there for a long time.

Rheumatologist in-training, RT1

They stay in their pyjamas or their gown. They assume the role of the patient and can potentially become...more passive and not as active, which we know is not helpful for back pain, so I think being in their own environment sooner is absolutely an advantage.

Physiotherapist, PH1

Avoiding unnecessary hospital admission was also seen as important for avoiding risks associated with unnecessary procedures (eg, spinal imaging), bed rest and immobility, acquiring infections, and other complications. For instance, a rheumatology trainee noted:

I think the recovery will be quicker once they’re at home and there’s less risk of…infections… patients will come in, they won’t mobilize much, they’ll get a chest infection.

Rheumatologist-in-training, RT1

While a rheumatologist summarized the issue as:

You don’t want to go to hospital because as people have pointed out, they’re full of sick people and you don’t particularly want to expose yourself to a lot of people being unwell or potential for infections. You’re also at some risk of reinforcing illness behaviour, and conviction that there is something serious wrong.

Consultant rheumatologist, RC8

#### Memory, Attention, and Decision Processes (Decision-Making)

Participants felt that for virtual health models to be successful, effective decision support and guidance were needed. This included access to both information about the virtual care option and support for patients who chose to take that option. With regard to the provision of information, it was suggested that intranet shortcuts, patient handouts, and posters in the ED would be useful in directing patients to virtual care and thereby avoiding unnecessary admissions. Patient-facing wording suggestions included:

Did you know we can manage this remotely? We can manage this virtually and you can be in comfort of your own home, but still have all the care that you need.

Physiotherapist, PH1

However, to implement such a system effectively, it was recognized that experienced clinicians were needed to monitor patients, provide them with reassurance, and effectively screen for serious pathology. Ideally, access to a clinician would be 24/7, and an escalation pathway would be in place in cases of deterioration or if a patient was not coping at home.

If this isn’t working out, there’s an option...there’s an escape clause…so the patient says look if I can’t cope, is there a number I can ring, and someone will pick me up? And take me to hospital again.

Consultant rheumatologist, RC8

Overall, care in the home was championed, with early supported discharge being regarded as the safest option to balance thorough evaluation with decreased risks of hospitalization. The benefits of care at home, with social support and a familiar environment, were promoted.

[It would] encourage the patient to be mobilising within an environment that they’re familiar with and feel safe in. They get the support of their family. And they will probably integrate what they learn…the physiotherapy and movements in their day-to-day lives much quicker.

Consultant rheumatologist, RC9

#### Beliefs About Capabilities (Perceived Competence, Professional Confidence, and Empowerment)

For patients to accept virtual care over hospital admission, participants felt it was important to build trust with patients through effective communication. In particular, it was important to ensure that patients did not feel they were being dismissed or a victim of a cost-cutting measure.

The important thing is how it’s framed to the patient, that we have a number of ways of potentially looking after you. And we believe that the one that’s going to result in the best outcomes for you would be to be looked after at home. So it’s about that framing and…providing concrete and appropriate reassurance.

Consultant rheumatologist, RC9

Participants expressed valuing a multidisciplinary team working collaboratively, with a focus on physiotherapy involvement to encourage patient mobility. To ensure patient safety, there was a strong preference for hybrid care combining virtual and home visits.

My preference would be to have a nurse visit the next day…then I would be much happier about the system…I would have a Physio go out the next day and/or maybe a nurse. But someone making sure they’re taking their medication. That they’re able to get to the toilet, and that they’re safe.

Consultant rheumatologist, RC2

I don’t think doctors have a great role to play in most back pain patients apart from firstly diagnosing the issue and excluding anything serious…we kind of leave it in the hands of the Physiotherapist to get people moving.

Consultant rheumatologist, RC3

## Discussion

### Overview

This study used the TDF to explore clinician views on barriers to and enablers of implementation of a virtual model of care for low back pain, which will inform efforts to reduce avoidable hospital admissions for back pain. By engaging stakeholders, the development of the virtual hospital model can be tailored to their specific needs and context, thereby increasing the likelihood of a successful implementation of a novel model of care [22,23]. Below, we explore some key barriers to implementation that arose from this study and potential solutions.

### Overcoming Challenges to Access

Our participants’ observations regarding the challenges associated with access and availability of virtual platforms are consistent with the literature [24-26]. It raises important questions regarding how the introduction of virtual models of care may inadvertently introduce new types of inequity. While virtual health services may improve access for some (eg, patients living in rural areas), for patients without internet access or who do not have sufficient digital literacy skills to avail themselves of these services, access may be compromised. Specific groups that are known to engage with virtual care less include cultural and ethnic minority groups, older people, and socioeconomically disadvantaged groups [27]. Physiotherapists delivering virtual musculoskeletal care during the pandemic have also reported challenges with delivering quality care in the case of poor internet quality, poor room setup (lighting, camera angles, and space), and low levels of patients’ technological skills [28].

A number of solutions to these concerns have been proposed, including having a technologically competent caregiver present during virtual sessions, simple email links to access services that do not require passwords or other configuration, simplified dashboards, and access to closed captioning if required for patients with hearing impairment [24]. Other strategies considered helpful for delivering care include ensuring access to a high-quality telehealth platform with a reliable internet connection and camera; providing patient resources (written or web-based information), videos or exercise apps, follow-up email summaries; and providing patient telehealth instructions ahead of the appointment [28]. We have incorporated all these suggestions into our model, and we intend to offer patients the option to loan internet-enabled devices to support effective and equitable delivery. It is estimated that 15% of patients eligible for our service would require interpreter services [13]. Therefore, we will use back pain care handouts translated into 10 key community languages to further support equitable access to health care information for CALD communities.

### Overcoming Concerns About Safety and Effectiveness

Our participants were comfortable with using virtual platforms, but they had reservations about the impact of these on their capacity to build patient rapport and trust and to complete a thorough examination. Clinicians were particularly concerned about missing serious pathologies, patients that present a high fall risk, and the safe management of analgesia at home. Participants also expressed potential difficulty managing patient and family expectations of traditional admission when presenting to the ED, as well as patients presenting with multimorbidity or who may be socially isolated. Some participants in senior roles shared concerns about the medicolegal liability of delivering virtual care should a safety issue arise.

Despite these concerns, evidence suggests that, when done well, virtual care for the treatment of lower back pain is not inferior to in-person treatment. While there can be increased challenges in the provision of virtual care, physical examinations of low back pain can be adapted effectively for the virtual environment [29]. It has also been shown that active listening can help uncover serious pathologies that may otherwise be overlooked [30]. A recent systematic review on the effectiveness and safety of telehealth for treatment of low back pain [31] suggests that remote clinical management is safe, effective, and does not compromise patient satisfaction. However, evidence is based on observational studies, and clinical trials aimed at optimizing telehealth delivery are needed.

Several strategies will be implemented in our Back@Home model of care to address these safety concerns, based on input provided by clinicians with experience in outpatient musculoskeletal virtual care [32]. First, referrals will only be accepted if the patient would otherwise have been admitted under the rheumatology or general medicine specialties. Patients with low back pain who would otherwise have been admitted under the neurosurgery or geriatrics teams are excluded from the program due to their higher risk profile and need for more intensive management [12]. Second, a clear escalation plan will be implemented for the clinically deteriorating patient, and police welfare checks will be conducted if both the patient and their next of kin are uncontactable. Third, to ensure appropriate weaning of stronger analgesia provided in the ED, all Back@Home patients are reviewed by a virtual hospital general practitioner, and a discharge summary is sent to the patient’s community general practitioner to ensure continuity of care and linking into outpatient services. Fourth, to build trust and rapport regular physiotherapy contact (daily if required) has been integrated into our model.

### Advantages of Virtual Care Despite Challenges

Despite multiple challenges, clinicians could see the potential benefits of delivering virtual care for acute back pain. These include health care cost-saving and improved patient flow, which are important considerations given the rising demands on public health care infrastructure and increased health spending. Participants also noted that there are benefits associated with avoiding hospital admission. These include reducing patients’ excessive dependence on the health care system, reducing inappropriate bedrest that could lead to deconditioning, and reducing the risk of hospital-acquired infection. These findings are supported by studies that have found that, for specific groups of patients, avoiding hospital admission provides similar benefits to patients as inpatient treatment [7].

### Strengths and Limitations

A strength of this study is the purposive sampling of rheumatologists, ED physicians, nurses, and physiotherapists likely to be involved in referring to the proposed virtual care model. A sample of transcripts was coded by 4 authors, adding to the richness of emerging themes.

However, clinicians were all based in urban tertiary care centers in 1 health district. Further investigation of barriers and enablers in other districts, including rural and regional centers, and in communities with more CALD individuals, would be helpful before implementation in those areas.

We were unable to include patient stakeholder perspectives due to COVID-19 pandemic-related hospital restrictions. For the next phase of our research, we have recruited patient participants and look forward to sharing their experience with virtual hospital care.

### Conclusion

The successful implementation of Back@Home relies on key stakeholder buy-in. Addressing barriers to implementation (where feasible) and building on enablers will be crucial for clinician adoption of this model of care. Based on clinicians’ input, the Back@Home model of care will incorporate the loan of internet-enabled devices, health care interpreters, home visits, and translated written resources in community languages to facilitate more equitable access to care for marginalized groups.

## Appendix

Multimedia Appendix 1 Interview guide.

## Abbreviations

CALD: culturally and linguistically diverse
COREQ: Consolidated Criteria for Reporting Qualitative Research
ED: emergency department
LHD: Local Health District
TDF: Theoretical Domains Framework

## Data Availability

The data sets generated during this study are available from the corresponding author on reasonable request.
