# Supplementary material for: Clinicians’ Perspective on Implementing Virtual Hospital Care for Low Back Pain: Qualitative Study
Source: JMIR Rehabil Assist Technol. 2023 Nov 21;10:e47227. doi: 10.2196/47227 (PMC10698644; doi:10.2196/47227)
Supplement: Multimedia Appendix 1 [file rehab_v10i1e47227_app1.docx]

**Interview guide:**

Thank you for volunteering your time today. Can you please confirm that you consent to this Zoom session being recorded and transcribed, to be accessed by the research investigating team only?

[If No, end session].

Can you please confirm that you have read the Participant Information Statement, understand what you have read, agree to take part in the research study as outlined in the statement, and agree to the use of your personal information as described?

[Record YES in data collection sheet ‘Consent’ column. If NO, end session].

1. How many years have you worked as a clinician?
2. What is your clinical role?
3. What are your thoughts on hospital admission for people with non-serious back pain?
4. What do you think are the main reasons people present to emergency with non-serious back pain and are subsequently admitted?
5. “There are plans to admit suitable patients with non-serious back pain to the new virtual hospital. Patients would be offered the opportunity to be admitted to the virtual hospital, receiving monitoring and care remotely. They would have regular nursing and medical review by video call, as well as video call sessions with a physiotherapist.”
6. What would be your concerns about being a virtual hospital physiotherapist for patients with back pain?
7. What do you think would be beneficial about being a virtual hospital physiotherapist for patients with back pain?
8. Would you like to share any other thoughts about potential virtual hospital care?

**Conclusion of interviews:**

Thanks again for your time and contribution to this research, it is very much appreciated.
